# Cooperation of Oncolytic Herpes Virotherapy and PD-1 Blockade in Murine Rhabdomyosarcoma Models

**DOI:** 10.1038/s41598-017-02503-8

**Published:** 2017-05-24

**Authors:** Chun-Yu Chen, Pin-Yi Wang, Brian Hutzen, Les Sprague, Hayley M. Swain, Julia K. Love, Joseph R. Stanek, Louis Boon, Joe Conner, Timothy P. Cripe

**Affiliations:** 1 Center for Childhood Cancer and Blood Diseases, Nationwide Children’s Hospital, The Ohio State University, Columbus, Ohio USA; 2 The Ohio State University College of Medicine, Columbus, Ohio USA; 3 Division of Hematology/Oncology/Blood and Marrow Transplantation, Nationwide Children’s Hospital, The Ohio State University, Columbus, Ohio USA; 4 Bioceros B.V., Utrecht, The Netherlands; 5 Virttu Biologics, Ltd, Glasgow, UK

## Abstract

Oncolytic virotherapy is an effective immunotherapeutic approach for cancer treatment via a multistep process including direct tumor cell lysis, induction of cytotoxic or apoptosis-sensitizing cytokines and promotion of antitumor T cell responses. Solid tumors limit the effectiveness of immunotherapeutics in diverse ways such as secretion of immunosuppressive cytokines and expression of immune inhibitory ligands to inhibit antitumor T cell function. Blocking programmed cell death protein (PD)-1 signaling, which mediates T cell suppression via engagement of its inhibitory ligands, PD-L1 or PD-L2, is of particular interest due to recent successes in many types of cancer. In syngeneic murine rhabdomyosarcoma models, we found that M3-9-M (MHC I high) but not 76-9 (MHC I low) tumors respond to oncolytic herpes simplex virus-1 (oHSV-1) and PD-1 blockade combination therapy. In addition, the therapeutic outcomes in M3-9-M tumor models correlated with the increased incidence of CD4^+^ and CD8^+^ T cells but not with the CD4^+^CD25^+^Foxp3^+^ regulatory T cell populations in the tumor. Overall, our data suggest the combination of PD-1 blockade and oHSV-1 may be an effective treatment strategy for childhood soft tissue sarcoma.

## Introduction

Oncolytic viruses were originally envisaged to be a therapeutic platform for cancer by virtue of their ability to preferentially kill tumor cells directly. The major barrier to their implementation was thought to be antiviral immunity, which would limit the spread and duration of the infection. There is now ample evidence suggesting that the immune response to oncolytic viruses can be therapeutic both via changes in the tumor microenvironment and the induction of antitumor T cell immunity^1^. Such effects, however, are likely subject to immunoevasive mechanisms characteristic of many cancers.

Solid tumors evade antitumor immunity by a variety of mechanisms including secretion of immunosuppressive cytokines, recruitment of suppressive immune cells and expression of T cell inhibitory ligands. The T cell exhaustion marker, PD-1, has emerged as an effective cancer therapeutic target, particularly for tumors that express its ligands PD-L1 and/or PD-L2^2^. Inhibitors of this axis are most effective in patients with cancers harboring high numbers of nonsynonymous genetic mutations and therefore expressing high levels of neoantigens^3, 4^. Whether antitumor T cells elicited in the context of intratumoral herpes simplex virus infection are subjected to the same suppressive effects, and thus might be enhanced by the same strategies, remains to be elucidated. In addition, the microenvironmental conditions that influence the outcome of viroimmunotherapy are poorly understood.

We recently exploited two explantable syngeneic mouse rhabdomyosarcoma tumor models to study herpes virus-induced T cell-mediated antitumor effects^5^. The first model, 76-9, is a methylcholanthrene-induced embryonal mRMS that was originally derived from a female C57BL/6 mouse^6^. The second model, M3-9-M, was derived from a male C57BL/6 mouse transgenic for hepatocyte growth factor and heterozygous for mutated p53^7^. We found that both models show significant response to oHSV virotherapy in C57BL/6 hosts, despite poor tumor susceptibility to oncolytic human HSV-1 infection and replication^5^. The effect was lost when these studies were conducted in athymic nude mice, which suggests this efficacy is dependent on an antitumor T cell response and thus might benefit from PD-1 inhibition. Phenotypic analysis revealed that indeed both mRMS cell lines expressed high levels of PD-L1^8^. We also found that while each displayed MHC class I, surface expression of this protein was considerably higher in M3-9-M than in 76-9^5, 8^. These models thus provide an ideal setting for investigating the response to T cell checkpoint inhibitors in combination with oncolytic herpes simplex virotherapy.

## Results

### Combining HSV1716 with anti-PD-1 antibody significantly prolongs survival in mice bearing M3-9-M tumors

**Figure 1:**
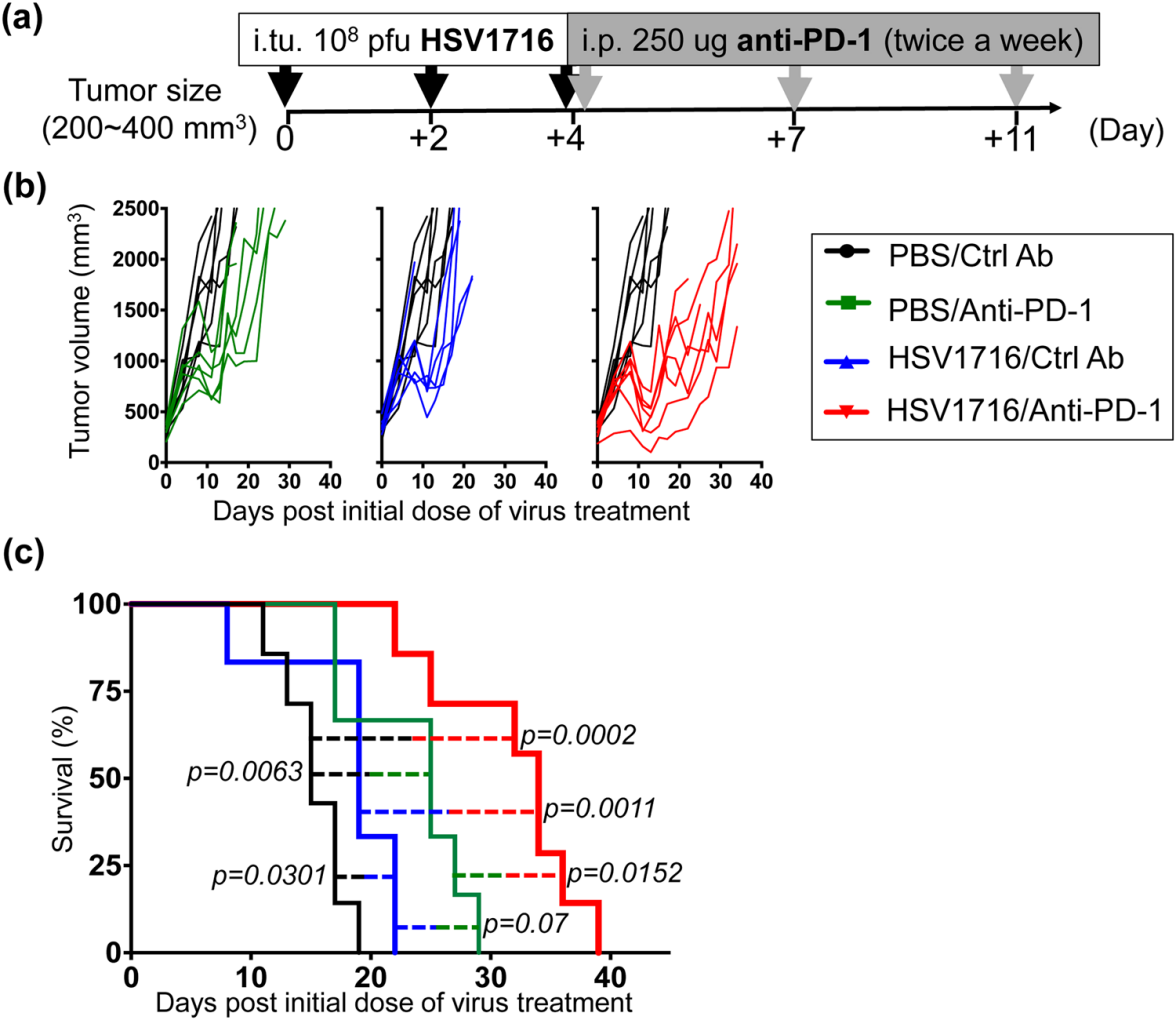
Combination of HSV1716 with anti-PD-1 antibody significantly prolongs survival in the male M3-9-M tumor model. (**a**) Schematic illustrates the dosing regimens for mice bearing subcutaneous M3-9-M tumors. Male C57BL/6 mice were implanted with 5 × 10^6^ M3-9-M cells subcutaneously and treated with three intratumoral (i.tu.) injections of 10^8^ pfu HSV1716 followed by intraperitoneal (i.p.) injections of anti-PD-1 antibody. (**b**) Tumor volumes of mice treated with anti-PD-1 (green lines; n = 6), HSV1716 (blue lines; n = 6) and combined therapy (red lines; n = 7) were measured twice a week and plotted individually against tumor volumes recorded for control mice (black lines; n = 7). (**c**) Kaplan-Meier survival curves for each treatment group demonstrate the improved efficacy of combining PD-1 blockade with HSV1716 virotherapy. Survival data were evaluated for statistical significance with Log-rank Mantel-Cox test.

We implanted male C57BL/6 mice with 5 × 10^6^ M3-9-M cells subcutaneously and allowed the tumors to reach a size of ~350 mm^3^ before initiating treatment (Fig. 1a). We then injected the tumors directly with the oncolytic virus HSV1716 (which was derived from an HSV-1 clinical isolate and is deleted for *RL1*, encoding ICP34.5^9^) or a vehicle control. Subsets of these mice were also regularly given intraperitoneal injections of PD-1 blocking antibody or an isotype control. Control animals displayed rapid tumor growth, reaching our endpoint criteria within 3 weeks (Fig. 1b and c, black line). Conversely, animals that were treated with PD-1 blocking antibody (Fig. 1b and c, green line) or intratumorally with HSV1716 (Fig. 1b and c, blue line) showed delayed tumor growth and enhanced overall survival. While the antitumor efficacy of these monotherapies was significant compared to the vehicle control, we found no statistical significance between the two. In contrast, their combination resulted in even greater antitumor efficacy and substantially prolonged overall survival compared to PD-1 blockade or HSV1716 treatments alone (Fig. 1b and c, red line).

We performed similar efficacy and survival studies in female C57BL/6 mice transplanted with 76-9 tumors, which express low levels of MHC class I compared to M3-9-M^5^. In contrast to our M3-9-M studies, the combination of anti-PD-1 and HSV1716 failed to significantly impact tumor growth and animal survival (Fig. S1).

### PD-1 blockade & HSV1716 combination therapy is more effective in the gender mismatched female M3-9-M tumor model

**Figure 2:**
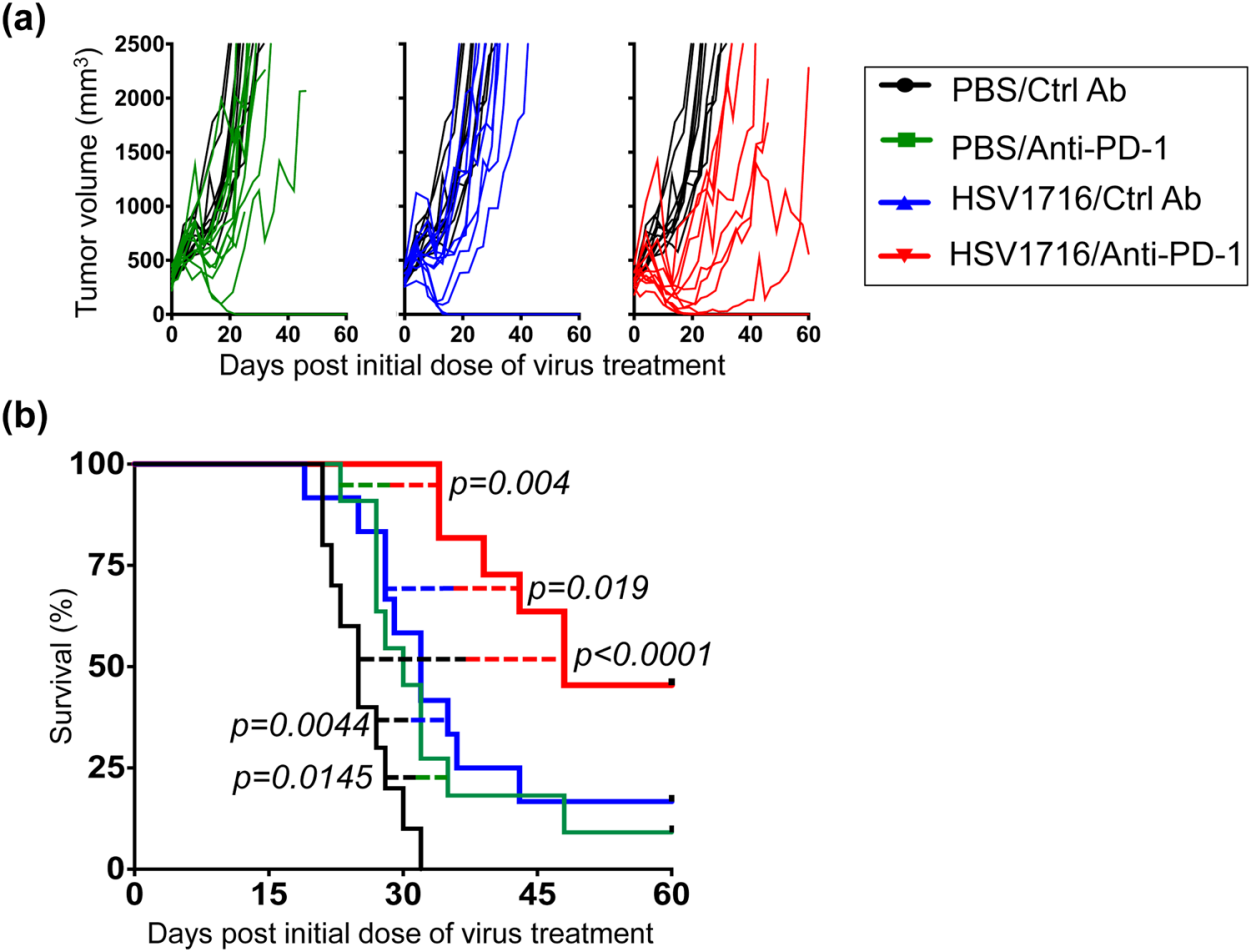
Combination of HSV1716 with anti-PD-1 antibody significantly prolongs survival with several complete responses in the more immunogenic female M3-9-M tumor model. Female C57BL/6 mice were implanted with 5 × 10^6^ M3-9-M cells subcutaneously and treated with three intratumoral injections of 10^8^ pfu HSV1716 followed by intraperitoneal injections of anti-PD-1 antibody as described in Fig. 1a. (**a**) Individual tumor volumes of mice treated with anti-PD-1 (green lines; n = 11), HSV1716 (blue lines; n = 12) and combined therapy (red lines; n = 11) plotted against tumor volumes recorded for control mice (black lines; n = 10). (**b**) Kaplan-Meier survival curves for each treatment group demonstrate the improved efficacy of combining PD-1 blockade with HSV1716 virotherapy in the more immunogenic female M3-9-M model. Mice deemed cured were subsequently re-challenged with M3-9-M injection on their contralateral flank and found to be resistant to new tumor growth (see Fig. S2). Survival data were evaluated for statistical significance with Log-rank Mantel-Cox test.

Previous studies have utilized male M3-9-M tumor cells implanted into female hosts to create an artificially “immunogenic” tumor model, due to expression of a foreign H-Y antigen^8^. In this model, PD-1 blockade was able to more effectively control tumor burden in female mice than their male counterparts^8^. To test if our combination therapy followed the same rule, we implanted male M3-9-M tumor cells into female C57BL/6 mice and examined the response to combination therapy (Fig. 2). Compared to the male model (Fig. 1), vehicle control tumors in female mice grew at a slower rate and took a longer time to reach endpoint criteria (Fig. 2a and b, black line). Animals who received PD-1 blocking antibody (Fig. 2a and b, green line) or HSV1716 (Fig. 2a and b, blue line) therapy alone showed delayed tumor growth to a greater extent than that exhibited by their male counterparts, which in some cases led to complete tumor regression. Combining PD-1 blockade with HSV1716 also resulted in a significantly better therapeutic response, with nearly 50% of treated animals surviving beyond 60 days post treatment (Fig. 2a and b, red line). All cured mice were resistant to M3-9-M tumor re-challenge, suggesting that these mice had developed tumor-specific memory T cells (Fig. S2).

**Figure 3:**
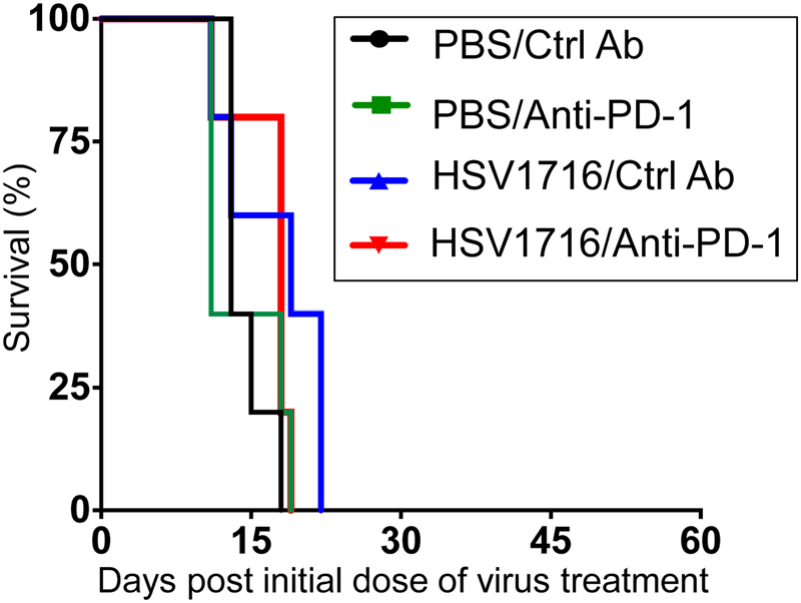
Combination therapy loses antitumor efficacy in nude mice. Female nude mice were implanted with 5 × 10^6^ M3-9-M cells subcutaneously and treated with three intratumoral injections of 10^8^ pfu HSV1716 followed by intraperitoneal injections of anti-PD-1 antibody as described in Fig. 1a. Kaplan-Meier survival curves for each treatment group (n = 5 per treatment group) demonstrate that combination therapy lost antitumor efficacy in nude mice. Survival data were evaluated for statistical significance with Log-rank Mantel-Cox test.

To confirm the contribution of adaptive immunity to PD-1 blockade and HSV1716 combination therapy, we repeated the same four-arm survival study using athymic nude mice (Fig. 3). As expected, PD-1 therapy was ineffective at slowing tumor growth in this setting, and likewise failed to enhance oncolytic virus-induced antitumor efficacy.

### PD-1 checkpoint inhibition does not significantly alter intratumoral viral kinetics

**Figure 4:**
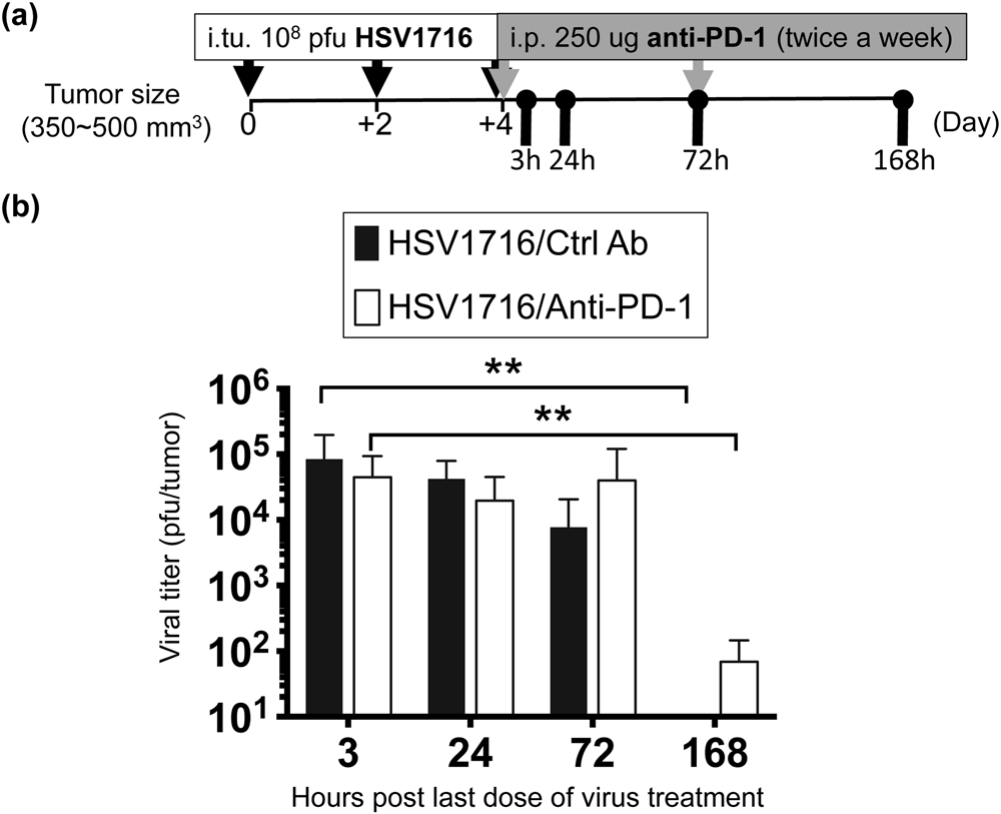
PD-1 blockade does not significantly alter intratumoral viral kinetics. (**a**) Schematic illustrates the dosing regimens and sample collection. Female M3-9-M tumor-bearing mice were treated with three doses of 1 × 10^8^ pfu of HSV1716 intratumorally (i.tu.) followed by intraperitoneal (i.p.) injection of anti-PD-1 or control antibody. Tumors were harvested 3, 24, 72 and 168 hours after last dose of HSV1716 injection for plaque assays. (**b**) Data are expressed as total plaque-forming units (pfu) per tumor with error bars representing SD (n = 6 per treatment group). Statistical analysis was performed by a one-way ANOVA with Tukey-adjusted *post hoc* tests (**p < 0.01).

In order to elucidate potential mechanisms behind the enhanced efficacy we observed following combination therapy, we initially examined the impact of PD-1 blockade on HSV1716 replication. We treated female mice bearing M3-9-M tumors as described in Fig. 1a and sacrificed them at different time points following the final dose of virus (Fig. 4a). We then recovered infectious virus particles from their tumors and quantified them via standard plaque assays. We noted no significant differences in the amount of recovered virus with or without PD-1 blockade (Fig. 4b). The presence of virus was lost over 168 hours in M3-9-M tumors regardless of other treatments, suggesting that PD-1 blockade does not significantly alter overall viral kinetics.

### Tumor microenvironments are more inflammatory when implanted into female mice

**Figure 5:**
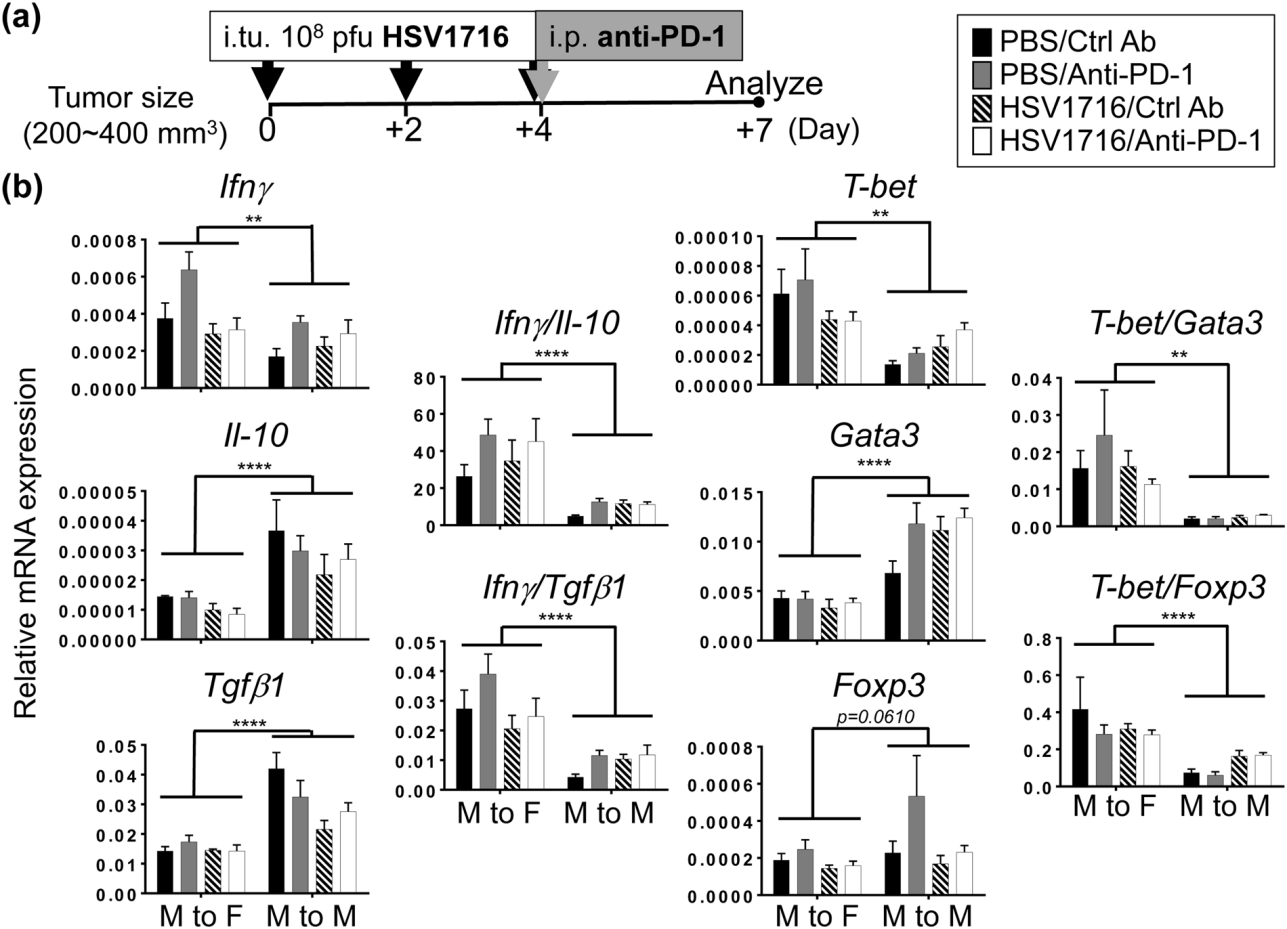
Tumors in female mice are more inflammatory than those in male mice. (**a**) Schematic illustrates the dosing regimens and sample collection. Female (M to F) or male (M to M) M3-9-M tumor-bearing mice received three doses of intratumoral (i.tu.) HSV1716 injection followed by intraperitoneal (i.p.) injection of anti-PD-1 or control antibody. Tumors were harvested 72 hours after last dose of HSV1716 injection. Th1 and Th2 related genes in tumors were evaluated by quantitative real-time PCR analyses. (**b**) Data show mean and SEM (n = 4~6 per treatment group). Statistical analysis was performed by a two-way ANOVA (*p < 0.05, **p < 0.01 and ***p < 0.001).

We next performed gene analysis by quantitative real-time PCR in an attempt to correlate therapeutic outcomes with the inflammatory status of the tumor microenvironment. We treated both female and male M3-9-M tumor-bearing mice with three intratumoral doses of HSV1716 followed by repeated intraperitoneal injections of anti-PD-1 or control antibody (Fig. 5a). We harvested tumor samples from each treatment group three days later to examine inflammatory and regulatory cytokine gene expression, but found no correlation between individual therapies and their cytokine expressions (Fig. 5b). We also did not find any correlation when we examined the expression of *T-bet* and *Gata3*, which are transcription factors associated with Th1 and Th2 adaptive immune responses, respectively (Fig. 5b). Interestingly, we found that M3-9-M tumors in female mice expressed higher levels of interferon gamma (*Ifnγ*) and lower levels of interleukin 10 (*Il-10*) and transforming growth factor beta 1 (*Tgfβ1*) than those in male mice (Fig. 5b). In addition, tumors harvested from female mice displayed relatively higher *T-bet* and much less *Gata3* mRNA compared to male mice. Taken together, these results suggest that male tumors grown in female mice elicit a stronger Th1 immune response than male mice, which might help trigger a stronger antitumor immune response to oHSV and PD-1 blockade therapy.

### PD-1 blockade augments oHSV-induced T cell infiltration in spleen

**Figure 6:**
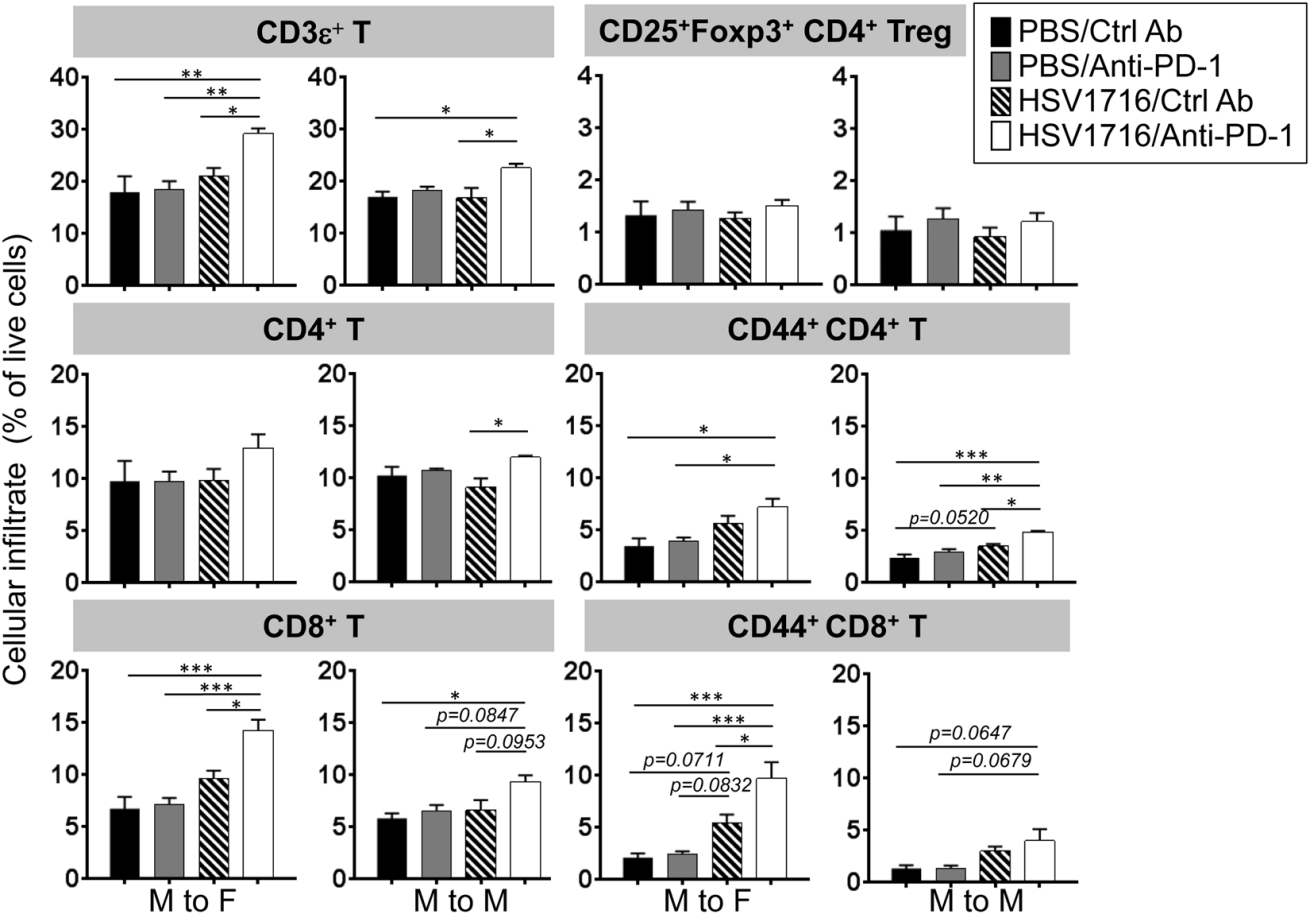
The splenic population of CD8^+^ T cells increases following combination therapy and varies with tumor immunogenicity. Female (M to F) or male (M to M) M3-9-M tumor-bearing mice received three doses of intratumoral HSV1716 injection followed by intraperitoneal injection of anti-PD-1 or control antibody as shown in Fig. 4a. Immune cell infiltrates in spleens were evaluated by flow cytometry analyses 72 hours after last dose of HSV1716 injection. Data show mean and SEM (n = 3~6 per treatment group). Statistical analysis was performed by a one-way ANOVA with Tukey-adjusted *post hoc* tests (*p < 0.05, **p < 0.01 and ***p < 0.001).

To determine whether these individual therapies or their combination had an influence on systemic immunity, we harvested spleens from both male and female M3-9-M tumor-bearing mice and compared their cell profiles. We found that none of the therapies had any significant impact on the composition of myeloid lineage cells (including neutrophils, monocytes and macrophages) in the spleens of either male or female mice (Fig. S3). We did note, however, that combination therapy led to an increase of splenic CD3ε^+^ T cells, which were slightly higher in the more immunogenic female M3-9-M model (Fig. 6). There was a significant increase in CD8^+^ T cells in female mouse spleens following combination therapy, which also produced a similar but less pronounced trend in the male mice. Combination therapy also led to slight increases in CD4^+^ T cell presence in spleens, but had no impact on the CD25^+^Foxp3^+^CD4^+^ sub-population of T regulatory (Treg) cells in either model (Fig. 6). We also examined the effects of these treatments on the CD44^+^ population of CD4^+^ and CD8^+^ T-cells, which constitute antigen-experienced or effector memory T cells. PD-1 blockade as a single agent had no effect on CD44 expression in either T cell population compared to the control group, but PD-1 blockade combined with virotherapy led to increased CD44^+^ CD8^+^ T cells, suggesting PD-1 blockade augments virus-induced Th1 immunity (Fig. 6). We were also able to detect anti-HSV CD8^+^ T cells in the spleen one week after the first dose of HSV1716 as determined by HSV glycoprotein B (gB)-tetramer staining (Fig. S4). The incidence of gB^+^ CD8^+^ T cells also increased following PD-1 blockade (Fig. S4). Taken together, these data demonstrate that while virotherapy alone is able to induce systemic T cell immunity, this activity can be further augmented by the addition of PD-1 blockade.

### Increased T cell infiltrates in tumors correlate with improved therapeutic outcomes following PD-1 blockade and HSV1716 combination therapy

**Figure 7:**
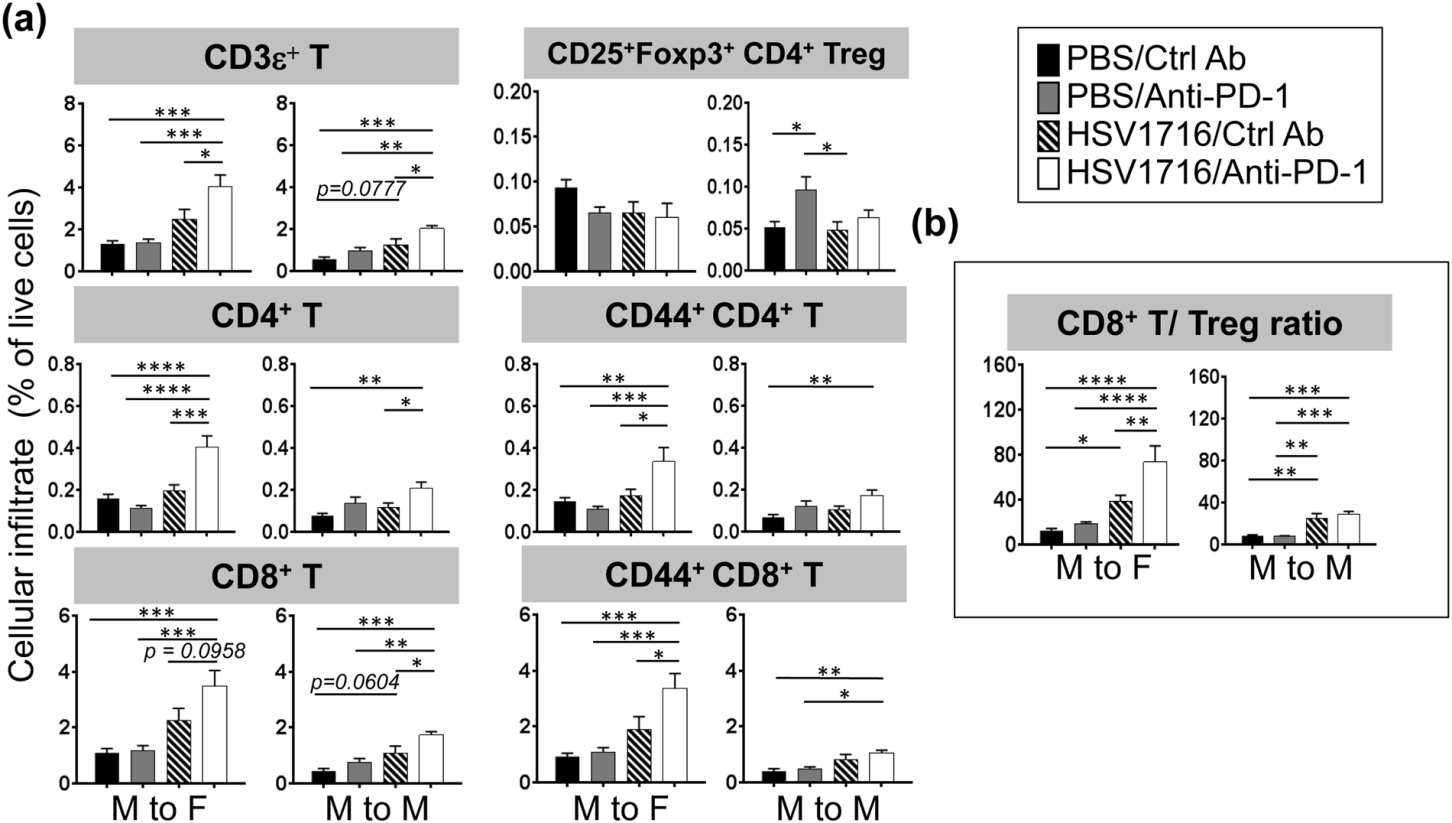
Combination therapy increases CD4^+^ and CD8^+^ T cells in tumor in proportion to immunogenicity. Female (M to F) or male (M to M) M3-9-M tumor-bearing mice received three doses of intratumoral HSV1716 injection followed by intraperitoneal injection of anti-PD-1 or control antibody as shown in Fig. 4a. (**a**) Immune cell infiltrates in tumors were evaluated by flow cytometry analyses 72 hours after last dose of HSV1716 injection. PD-1 blockade alone had little to no impact on overall T cell numbers, but enhanced HSV1716-induced T cell recruitment, particularly in the more immunogenic female M3-9-M model. (**b**) Combination therapy results in higher CD8^+^ T cell to Treg ratios in female M3-9-M model, which is associated with improved therapeutic outcomes. Data show mean and SEM (n = 5~8 per treatment group). Statistical analysis was performed by a one-way ANOVA with Tukey-adjusted *post hoc* tests (*p < 0.05, **p < 0.01, ***p < 0.001 and ****p < 0.0001).

To further elucidate the potential cellular actions of combination therapy locally, we harvested tumor samples from the different treatment groups and examined both innate and adaptive T cell immune cell populations. Applying single agent or combination treatment resulted in a small but statistically significant effect on the presence of some myeloid lineage cells in M3-9-M tumors, but the effects were not consistent between male and female mice (Fig. S5). Regarding lymphocyte cell populations, we found that PD-1 blockade by itself had little impact on T cell recruitment (Fig. 7a). HSV1716, in contrast, was able to induce more CD3ε^+^ T cell recruited to tumors in both models, particularly in the female mice. The incidence of T cells were further enhanced by combination therapy, in which we also observed the highest incidence of total CD8^+^ T cells and CD44^+^CD8^+^ T cells.

Compared to the control groups, combination therapy did not significantly change CD25^+^Foxp3^+^ Treg cells in the tumors regardless of the host gender. Instead, we found that combination therapy led to an increase of CD44^+^ CD4^+^ T cells, particularly in the female mice (Fig. 7a). When we compared the ratios of CD8^+^ T cells to Tregs (a metric associated with positive therapeutic responses^10^), we found that combination therapy was significantly higher than either monotherapy in the female mice (Fig. 7b). Taken together, these results suggest that combination therapy induces a shift in the effector T/Treg balance towards antitumor immunity (particularly in female mice), which likely contributes to superior antitumor responses.

Based on the observation that the presence of therapeutic virus was lost rapidly within a week post treatment (Fig. 4b), we attempted to enhance the combination therapy in male M3-9-M tumor models by repeated administration of virus together with anti-PD-1 blocking antibody. While this strategy trended towards improved therapeutic outcomes compared to the standard three doses of virus followed by anti-PD-1 antibody therapy, it was still insufficient to promote durable responses in the male M3-9-M model (Fig. S6).

## Discussion

Here we demonstrated that combining oHSV therapy with PD-1 blockade significantly enhanced overall survival in MHC I high M3-9-M murine rhabdomyosarcoma tumors. The efficacy of this approach was not dependent on enhanced viral kinetics or oncolytic activity, but rather appears to be due to the induction and/or maintenance of a localized antitumor T cell immune response. Anti-PD-1 and PD-L1 agents are among the most evaluated checkpoint inhibitors in both preclinical and clinical studies. Blocking this axis can prevent T cell exhaustion and therefore promote antigen-experienced T cell function. In this study, we found that anti-PD-1 antibody alone did not significantly alter overall T cell numbers in the tumors or spleens of treated mice. We did find, however, that intratumoral injection of HSV1716 could promote both a localized and a systemic increase of CD44^+^ memory T cells (CD4^+^ and CD8^+^). Combining HSV1716 with anti-PD-1 therapy further increased these populations of CD44^+^ T cells, leading to greater therapeutic responses. This observation held particularly true of the more immunogenic female host M3-9-M model, suggesting that tumors expressing strong tumor antigens may benefit more from this combination approach.

Our data suggest that the function of PD-1 blockade in the combination setting is to augment or sustain native or virotherapy-induced Th1 immunity against the virus and/or the tumor. Accordingly, we noted that the efficacy of our treatment was greatly diminished when we performed survival studies in athymic nude mice (Fig. 3). Likewise, Meadors *et al*. recently showed that mice pre-immunized with irradiated M3-9-M whole cell lysates were resistant to subsequent tumor challenge^7^. This antitumor effect could be partially inhibited by the depletion of either CD4^+^ or CD8^+^ T cells, or completely abolished by the depletion of both. This observation suggests that functional CD4^+^ and CD8^+^ T cells act in concert to strengthen the antitumor effect in this model.

Using the same M3-9-M tumor model with anti-PD-1 antibody administration and tumor cell implantation on the same day, Highfill *et al*. showed that PD-1 blockade controlled tumor growth much better in the more immunogenic female mice setting than in male mice^8^. We observed a similar pattern with the combination of anti-PD-1 and HSV1716. Although we attribute the difference in therapeutic outcomes between the genders to be due to the expression of the strong H-Y antigen on M3-9-M cells (which are immunologically foreign to female mice), we cannot rule out a role for sexual dimorphism in immune function. In the genetic absence of PD-1, Tregs in female mice function less well than those in male mice, and have been shown to result in more potent antitumor immunity in a melanoma model^11^. Likewise, the Tregs of female mice tend to have lower PD-1 expression than their male counterparts, which consequently makes them more sensitive to PD-1 blockade^11^. Thus, it is possible that some of the differences we observed could be related to gender differences in the tumor microenvironment. Our attempt to enhance antitumor efficacy in the male M3-9-M model by giving them repeated injections of HSV1716 ultimately proved to be insufficient (Fig. S6). Instead, given their higher expression of *Il-10* and *Tgfβ1*, a more rational approach might be to add immune modulators to bolster our current combination therapy by further reducing tumor immunosuppression.

The combination of HSV1716 with PD-1 blockade also did not make a significant impact on the 76-9 tumor model. Our previous study revealed that the 76-9 and M3-9-M tumor models are quite different^5^, suggesting multiple factors are involved in the therapeutic outcomes of HSV1716 and anti-PD-1 therapy. In addition to higher MHC I expression, M3-9-M tumors express more G-CSF and IFNγ compared to 76-9^5^. M3-9-M tumors also recruit more neutrophils, which Highfill *et al*. recently showed can antagonize PD-1 blockade therapy^8^. Although M3-9-M and 76-9 tumors recruit a similar amount of CD3ε^+^ T cells at baseline, the ratio of CD4/CD8 is higher in 76-9 (approximately 1.4) than those in M3-9-M (approximately 0.2)^5^. While further study is needed, it is possible that a greater proportion of these CD4^+^ T-cells in the 76-9 mRMS model are Treg cells, which can negatively control CD8^+^ T cells and restrict CD8^+^ T cell response to immunotherapy.

The effects of combined checkpoint inhibitors with oncolytic viruses are not limited to the herpes virus or to rhabdomyosarcoma that we tested here. Shen *et al*. showed improved antileukemia efficacy using a systemically administered oncolytic vesicular stomatitis virus in combination with anti-PD-L1 antibody therapy^12^. The effect was lost with depletion of NK and CD8^+^ T cells but not CD4^+^ cells. Woller *et al*. tested an oncolytic adenovirus in a mouse model of lung adenocarcinoma and found that virotherapy and anti-PD-1 combination increased the breadth and magnitude of antitumor T cells^13^. Expression of checkpoint inhibiting antibodies as transgenes improved the effects of an oncolytic measles virus in melanoma models^14^, associated with an increased CD8^+^/Foxp3^+^ immune cell ratio in tumors. Hardcastle *et al*. have also recently demonstrated that combining measles virus with anti-PD-1 blocking antibody enhanced antitumor efficacy with increased T cell influx into murine glioblastoma^15^. Rajani *et al*. found anti-PD-1 combined with reovirus in a melanoma model had pleomorphic effects, including enhanced NK killing of virus-infected cells, increased CD8^+^ T cells, and reduced Tregs^16^. Using a non-replicative Semliki Forest virus vector that expresses IL-12 in melanoma and colon cancer models, Quetglas *et al*. found that PD-L1 was induced on infected cells and combination with PD-1 or PD-L1 antibodies improved efficacy^17^.

To conclude, we provide preclinical data to support the translation of combination of anti-PD-1 and oHSV therapy strategy into clinical development. Our data suggest the combination of PD-1 blockade and oncolytic herpes virotherapy may be an effective treatment strategy, perhaps even more so for MHC I high expressing tumors. Future studies will focus on exploiting strategies to reduce immunosuppression in the tumor microenvironment to enhance oHSV and anti-PD-1 combination therapy.

## Material and Methods

### Cell lines

Two established murine rhabdomyosarcomas (RMS) cell lines, M3-9-M (transgenic for expression of hepatocyte growth factor/scatter factor and p53-/-) and 76-9 (methylchloranthrene-induced), were kindly provided by Dr. Crystal Mackall (Stanford University) and Dr. Brenda Weigel (University of Minnesota), respectively. Murine RMS cell lines were cultured in RPMI containing 15% heat inactivated fetal bovine serum (FBS), 1% L-glutamine, 1% non-essential amino acids, 50 μM 2-mercaptoethanol, 100 IU/mL penicillin and 100 μg/mL streptomycin at 37 °C in 5% CO_2_. Vero cells (American Type Culture Collection, Manassas, VA), were cultured in EMEM with 10% FBS and penicillin/streptomycin. All cell lines used in the study were tested free of mycoplasma using MycoAlert™ Mycoplasma Detection Kit (Lonza, Inc., Allendale, NJ).

### Animal studies

Animal studies were approved by the Nationwide Children’s Hospital Institutional Animal Care and Use Committee (IACUC; protocol number AR12-00074), and performed in accordance with guidelines established by NIH Guide for the care and use of laboratory animals. To establish tumors, 5 × 10^6^ of murine rhabdomyosarcoma cells were injected subcutaneously into the flanks of 6~8 week old C57BL/6 or athymic nude mice (Envigo, Frederick, MD). Tumor sizes were measured twice weekly by caliper after implantation and tumor volume was calculated by length × width^2^ × π/6, as described in our previous publication^5^. When tumors reached 200~400 mm^3^ in size, animals were pooled and randomly divided into four groups with comparable average tumor size. Mice were given fractionated HSV1716 (1 × 10^8^ pfu in 100 μL PBS) or 100 μL PBS injections every other day for a total of 3 injections. 250 μg of anti-PD-1 (RMP1-14, Bio-X-cell, West Lebanon, NH) or control antibody (2A3, Bio-X-cell) was given intraperitoneally twice a week for four weeks, beginning with the last dose of HSV1716 injection. For survival studies, animals were monitored for tumor volumes two times per week for 60 days after initial treatment, until tumor volume exceeded 2500 mm^3^, or until tumor diameter reached 2 cm. For cell recruitment analysis and *in vivo* gene expression, tumors were harvested 3 days after last dose of HSV1716 injection. All experimental groups were treated and measured in an unblinded manner.

### *In vivo* virus replication

M3-9-M tumors were established in 6~8 week old female C57BL/6 as described above. When tumors reached 350~500 mm^3^ in size, they received unfractionated HSV1716 (10^8^ pfu in 100 μL PBS) injections every other day for a total of 3 doses, followed by intraperitoneal anti-PD-1 or control antibody injection twice a week starting from the last dose of HSV1716 injection. At 3, 24, 72 and 168 hours post last dose of HSV1716 injection, tumor samples were harvested, freeze-thawed three times and the lysates were titrated by standard plaque assay on Vero cells.

### RNA isolation and gene expression

Tumors from mice were flash frozen and pulverized. Total RNA was isolated from tumor powder using the RNeasy Plus Mini Kit (Qiagen, Germantown, MD) according to the manufacturer’s instructions. RNA quantity and purity was determined using a NanoDrop 2000 Spectrophotometer (Themo Fisher Scientific, Charlotte, NC). 5 μg of total RNA was used to synthesize cDNA using SuperScript II Reverse Transcriptase (Life Technologies, Carlsbad, CA) according to the manufacturer’s instructions. Quantitative real-time PCR was performed using the Applied Biosystems 7900. iTaq universal SYBR green supermix kit (Bio-Rad, Hercules, CA) were used to quantify the gene transcripts in 10 ul reactions according to the manufacturer’s instructions. Cycling conditions included the initial step of 2 minutes at 50 °C and 5 minutes denaturation at 95 °C, followed by 40 thermal cycles of denaturation at 95 °C for 15 seconds, annealing at 58 °C for 30 seconds, and elongation at 72 °C for 30 seconds. The comparative quantitation method was used and the results are represented as fold gene expression relative to *Gapdh*: 2^−(C^_t_^target gene − C^_t_^*Gapdh*)^. The primers used in this study are listed in Table S1.

### Flow cytometry

Splenocytes were prepared by smashing spleen through a 70 μM cell strainer in PBS with a sterile 5 ml syringe plunger. Single tumor cell suspensions were obtained as described previously^5^. Single cell suspensions from both spleens and tumors were lysed with ACK RBC lysis buffer (Lonza, Inc.) and blocked with 5% mouse Fc blocking reagent (2.4G2, BD Biosciences, San Jose, CA) in FACS buffer (1% FBS and 1 mM EDTA in PBS). Cells were labeled with one of the following antibody staining panels for analysis of the innate and adaptive immune cells: (1) NK1.1-PE (PK136), CD11b-Violet 421 (M1/70), F4/80-PE-Cy7 (BM8), Ly6C-APC (AL-21), Ly-6G-APC-Cy7 (1A8); (2), CD4-APC (GK1.5), CD25-PE (7D4), CD8a-PE-Cy7 (53-6.7), CD3ε- Violet 421 (145-2C11), NK1.1-PerCP and B220-APC-Cy7 (RA3-6B2); (3) CD4-FITC, CD44-PE-Cy7 (IM7), CD8a-PE, CD3ε-Violet 421, NK1.1-PerCP and HSV-gB-TETRAMER-APC. HSV glycoprotein B (gB) monomer (SSIEFARL) was obtained from the NIH tetramer core facility (Human B2M H-2K^b^). To produce fluorochrome-conjugated HSV-gB tetramer, gB monomer was tetramerized using streptavidin-APC (SA1005, Molecular Probes, Eugene, OR) at a ratio of 1:3 monomer to streptavidin-APC. 10% of the total volume of streptavidin-APC was added to the monomer every 15 minutes up to 2 hours on ice. Single samples were stained with the above staining panels for 30 minutes on ice and washed one time with FACS buffer. After labeling, cells were fixed in 1% paraformaldehyde and a minimum of 100,000 events were collected and analyzed on a BD FACS LSR II (BD Biosciences). Analysis was carried out using the FlowJo software, version 10.0.3 (Tree Star Inc., Ashland, OR). For Foxp3 intra-cellular staining, mononuclear cells were enriched by Percoll (GE Healthcare Bio-Sciences, Pittsburgh, PA) density gradient centrifugation. Single cells were re-suspended in 6 mL of 44% Percoll and gradually loaded on top of 4 mL of 67% Percoll in a 15 mL tube. Samples were spun at 500 g without brake for 20 minutes at room temperature. The interface cells between 44% and 67% Percoll were collected, washed one time with FACS buffer and blocked with 5% mouse Fc blocking reagent in FACS buffer. Mononuclear cells were stained with cell surface markers including CD4-APC, CD8-PE-Cy7, CD25-PE, NK1.1-PerCP, B220-APC-CY7 and CD3ε-Violet 421 followed by Foxp3-FITC (FJK-16s) intracellular staining using cell fixation and cell permeabilization kit (Invitrogen GAS001S100 and GAS002S100, Themo Fisher Scientific). Stained cells were fixed in 0.5% paraformaldehyde but otherwise collected and analyzed as described above. All the staining antibodies were purchased from BioLegend (San Diego, CA) except for anti-Foxp3 (eBiosciences, San Diego, CA), anti-CD25 (BD Biosciences) and anti-Ly6C (BD Biosciences).

### Statistical analysis

Statistical analysis was conducted using the GraphPad Prism software (GraphPad Software, Inc., San Diego, CA). Kaplan-Meier curves and corresponding log-rank Mantel-Cox tests were used to evaluate the statistical differences between groups in survival studies. To assess differences in *in vivo* virus replication and cell recruitment among four treatment groups, one-way ANOVA with Tukey-adjusted *post hoc* tests were used. Two-way ANOVA was used to compare gene expression in male and female mouse models across all treatment groups.

## Electronic supplementary material

Supplementary information
