# Supplementary material for: Cooperation of Oncolytic Herpes Virotherapy and PD-1 Blockade in Murine Rhabdomyosarcoma Models
Source: Sci Rep. 2017 May 24;7:2396. doi: 10.1038/s41598-017-02503-8 (PMC5443787; doi:10.1038/s41598-017-02503-8)
Supplement: Supplementary file 1 — Supplementary information [file 41598_2017_2503_MOESM1_ESM.pdf]

Supplementary information

Cooperation of Oncolytic Herpes Virotherapy and PD-1 Blockade in Murine Rhabdomyosarcoma Models

Chun-Yu Chen[1], Pin-Yi Wang[1], Brian Hutzen[1], Les Sprague[3], Hayley M. Swain[1], Julia K. Love[1], Joseph R. Stanek[2], Louis Boon[4], Joe Conner[5], and Timothy P. Cripe[1,2]

[1]Center for Childhood Cancer and Blood Diseases, Nationwide Children's Hospital and [2]Division of Hematology/Oncology/Blood and Marrow Transplantation, Nationwide Children's Hospital, The Ohio State University, Columbus, Ohio, USA; [3]The Ohio State University College of Medicine, Columbus, Ohio, USA; [4]Bioceros B.V., Utrecht, The Netherlands; [5]Virttu Biologics, Ltd, Glasgow, U.K.

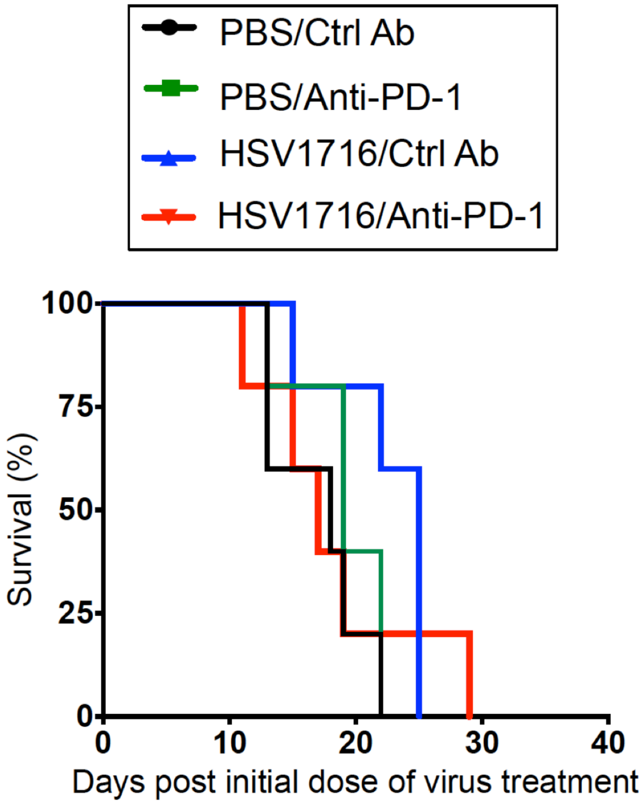

Figure S1. Combination therapy fails to improve antitumor efficacy in the 76-9 mRMS model. Female C57BL/6 mice were implanted with 5 x 10^6 76-9 cells subcutaneously and treated with three intratumoral injections of 10^8 pfu HSV1716 followed by intraperitoneal injections of anti-PD-1 antibody as described in Fig. 1a. Kaplan-Meier survival curves demonstrate that combination therapy had no impact on survival in the 76-9 mRMS model (n=5 per treatment group). Survival data were evaluated for statistical significance with Log-rank Mantel-Cox test.

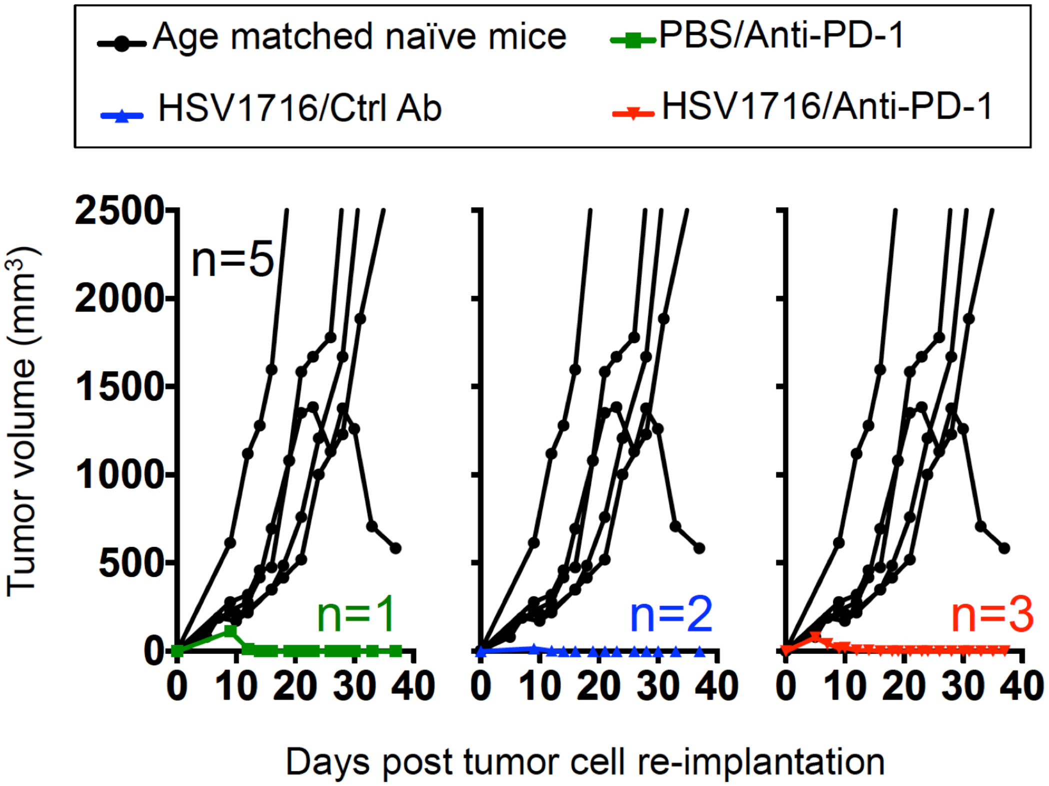

Figure S2. Cured mice demonstrate antitumor immunity. Female mice determined to be tumor-free after anti-PD-1 (green line; n=1), HSV1716 (blue lines; n=2) and combined therapy (red lines; n=3) were rechallenged with 5 x 10^6 M3-9-M cells subcutaneously. Naïve aged matched female mice (black lines; n=5) were challenged with 5 x 10^6 M3-9-M as a control. Tumor volumes were measured twice a week. None of the previously cured mice developed tumors.

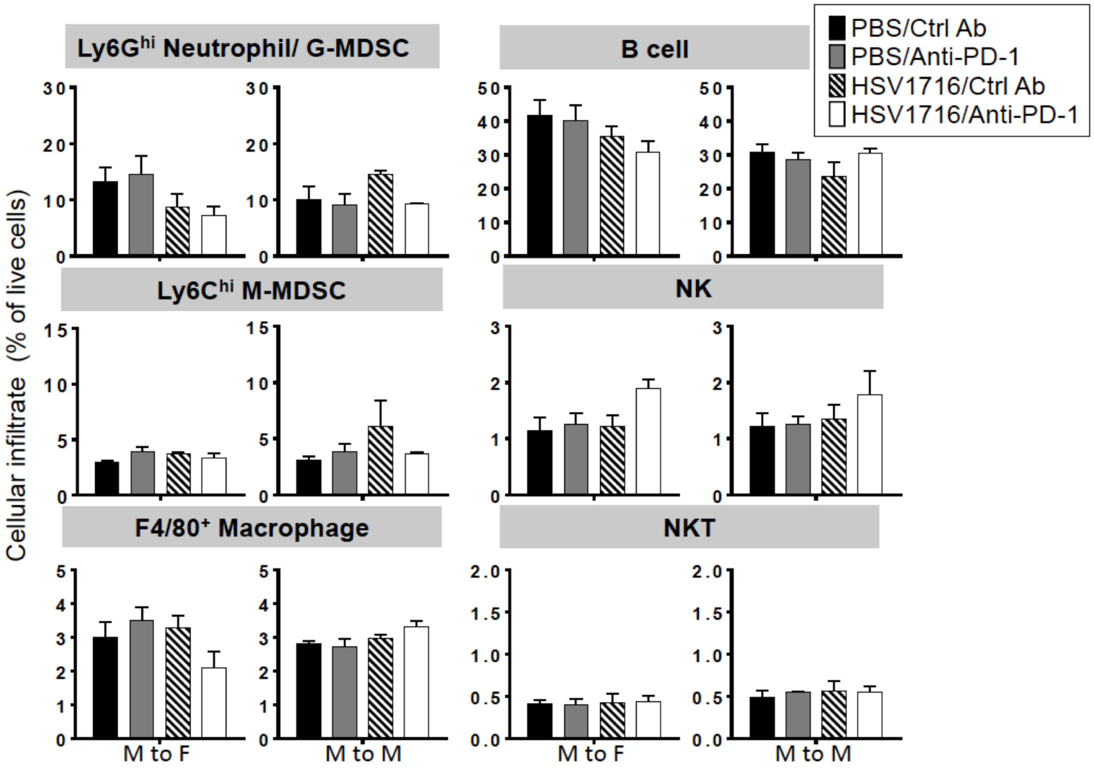

Figure S3. Combination therapy has no effect on myeloid cells, B cells, NK cells, or NKT cells in spleen. Female (M to F) or male (M to M) M3-9-M tumor-bearing mice received three doses of intratumoral HSV1716 injection followed by intraperitoneal injection of anti-PD-1 or control antibody as shown in Fig. 4a. Immune cell infiltrates in spleens were evaluated by flow cytometry analyses 72 hours after last dose of HSV1716 injection. Data show mean and SEM (n = 3~5 per treatment group). Statistical analysis was performed by a one-way ANOVA with Tukey-adjusted *post hoc* tests. Neutrophil: $CD11b^{+}Ly6c^{lo}Ly6g^{hi}$. M-MDSC: $CD11b^{+}Ly6c^{hi}Ly6g^{lo}$. Macrophage: $CD11b^{+}F4/80^{+}$. B cell: $CD3\varepsilon^{-}B220^{+}$. NK cell: $CD3\varepsilon^{-}NK1.1^{+}$. NKT cell**:** $CD3\varepsilon^{+}NK1.1^{+}$.

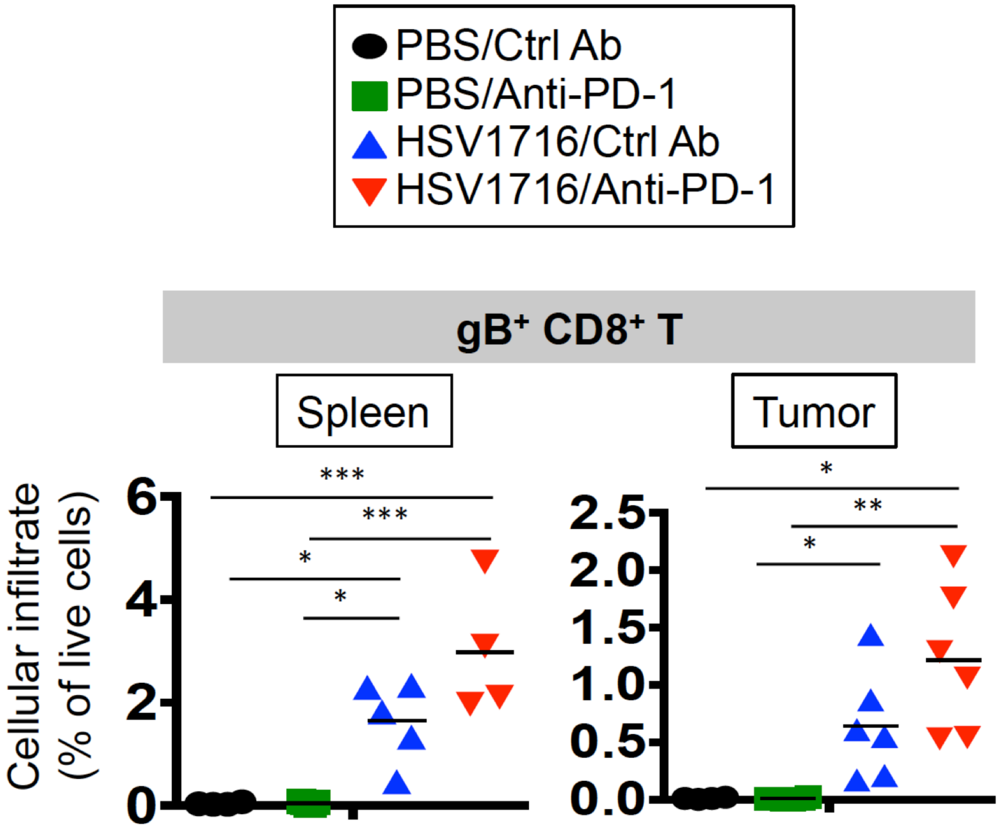

Figure S4. Intratumoral HSV1716 injection induces anti-virus $CD8^+$ T cells in spleen and tumor. Female M3-9-M tumor-bearing mice received three doses of intratumoral HSV1716 injection followed by intraperitoneal injection of anti-PD-1 or control antibody as shown in Fig. 4a. Dot plots show the percentage of gB^+ $CD8^+$ T cells in spleens and tumors 72 hours after last dose of HSV1716 injection (n= 4~6 per treatment group). Statistical analysis was performed by a one-way ANOVA with Tukey-adjusted *post hoc* tests (*$p < 0.05$, **$p < 0.01$ and ***$p < 0.001$).

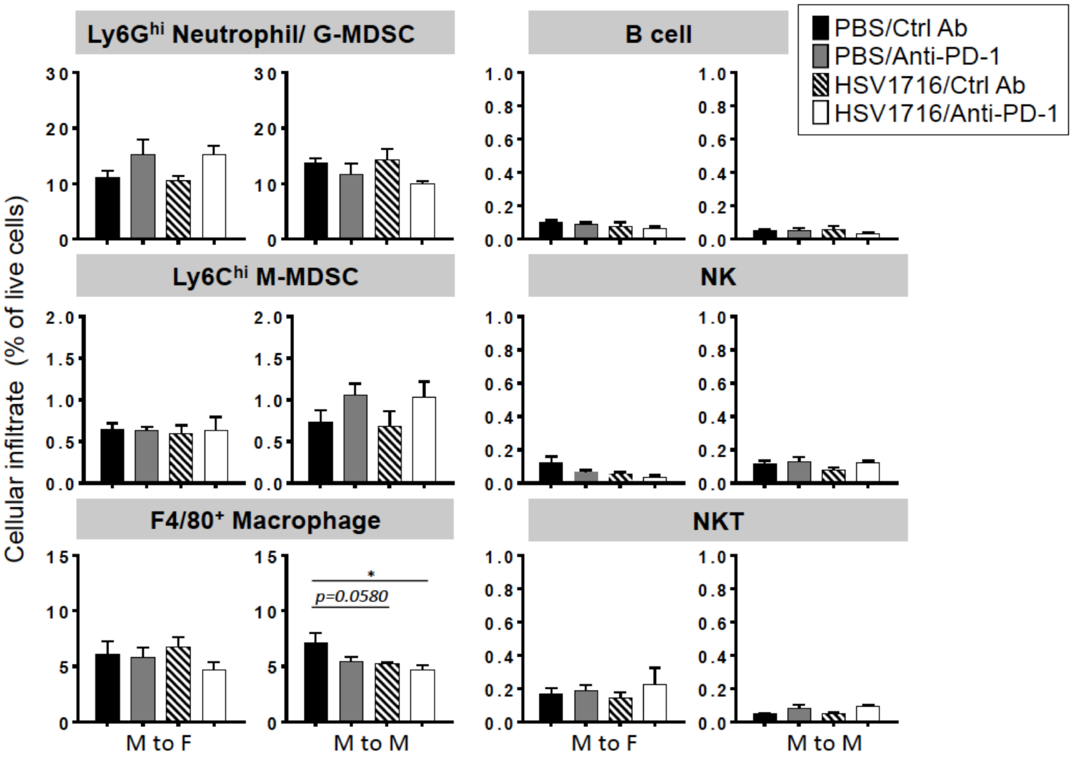

Figure S5. Combination therapy has minimal effect on myeloid cells in tumor. Female (M to F) or male (M to M) M3-9-M tumor-bearing mice received three doses of intratumoral HSV1716 injection followed by intraperitoneal injection of anti-PD-1 or control antibody as shown in Fig. 4a. Immune cell infiltrates in tumors were evaluated by flow cytometry analyses 72 hours after last dose of HSV1716 injection. Data show mean and SEM (n = 3~8 per treatment group). Statistical analysis was performed by a one-way ANOVA with Tukey-adjusted *post hoc* tests (*$p < 0.05$).

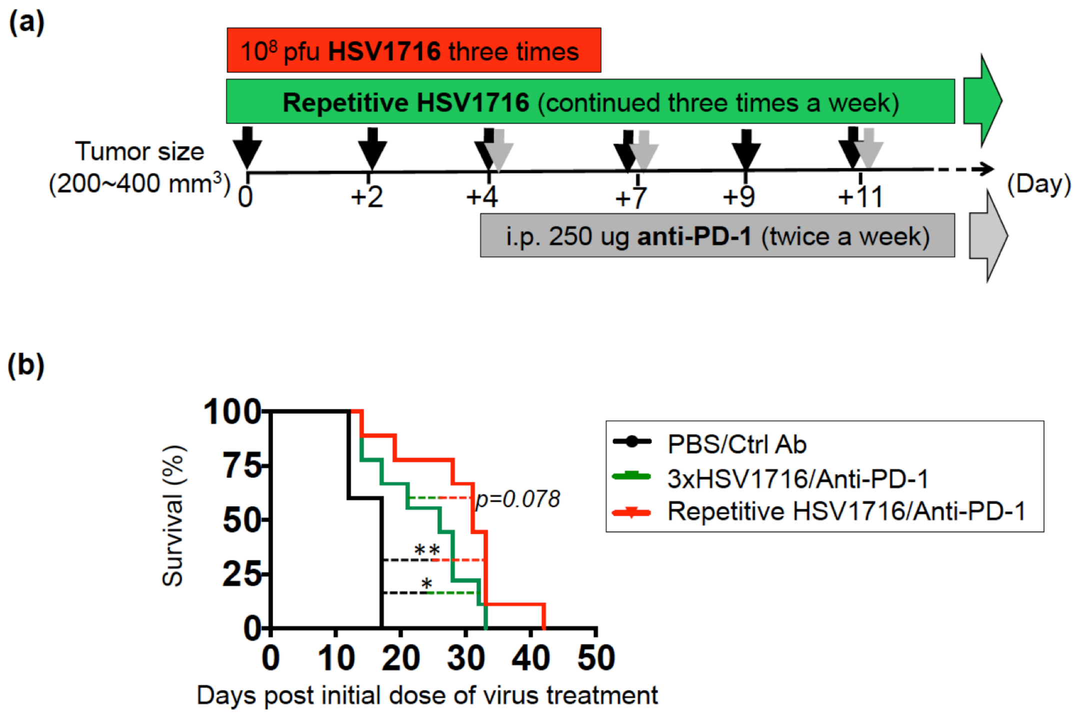

Figure S6. Repetitive HSV1716 injection moderately enhanced the antitumor efficacy of anti-PD-1 therapy compared to three doses of HSV1716 injections. (a) Schematic illustrates the dosing regimens for mice bearing subcutaneous M3-9-M tumors. Male C57BL/6 mice were implanted with 5 x 10^6 M3-9-M cells subcutaneously and treated with three intratumoral injections of 10^8 pfu HSV1716 as shown in Fig. 1a or repeated intratumoral injections of 10^8 pfu HSV1716 as shown here, followed by intraperitoneal injections of anti-PD-1 antibody. Tumor volumes of mice treated with three doses of HSV1716 and anti-PD-1 (green lines; n=9) or repetitive HSV1716 + anti-PD-1 (red lines; n=9) were measured twice a week and compared individually against tumor volumes recorded for control mice (black lines; n=5). **(b)** Kaplan-Meier survival curves for each treatment group were shown. Survival data were evaluated for statistical significance with Log-rank Mantel-Cox test (*$p < 0.05$ and **$p < 0.01$).

Supplementary Table 1: *Real-Time PCR primer sets*

	Forward Primer (5'-3')	Reverse Primer (5'-3')
Gapdh	TGCACCACCAACTGCTTAGC	GGCATGGACTGTGGTCATGAG
Ifnγ	TGATGGCCTGATTGTCTTTCAA	GGATATCTGGAGGAACTGGCAA
Il-10	ACTTGGGTTGCCAAGCCTTA	GGGGAGAAATCGATGACAGC
Tgfβ1	GCTGAACCAAGGAGACGGAAT	GCTGATCCCGTTGATTTCCA
T-bet	GGTGTCTGGGAAGCTGAGAG	GAAGGACAGGAATGGGAACA
Gata3	AGTGTGTGAACTGCGG	CCCCATTAGCGTTCCT
Foxp3	GGAGAAGCTGGGAGCTATGC	GTGGCTACGATGCAGCAAGA
